# Nano Zero-Valent Iron and Its Modifications for the Remediation of Heavy Metal-Contaminated Soils from Zinc–Lead Mine Areas in China

**DOI:** 10.3390/toxics14100861

**Published:** 2026-09-28

**Authors:** Wenbing Ji, Meng Chen, Zhewei Hu, Xiaoyu Zhang, Caiyi Zhao, Yanhong Feng, Qi Li, Tianning Li, Rongrong Ying, Aijing Yin

**Affiliations:** 1 Nanjing Institute of Environmental Sciences, Ministry of Ecology and Environment, Nanjing 210042, China; 2 Key Laboratory of Soil Environmental Management and Pollution Control, Ministry of Ecology and Environment, Nanjing 210042, China

**Keywords:** nano zero-valent iron, stabilizer modification, heavy metals, stabilization, contaminated soil, leaching toxicity

## Abstract

Nano zero-valent iron (nZVI) and its modified forms are promising for remediation of heavy metal-contaminated soils, but bare nZVI aggregates and oxidizes readily. In this study, carboxymethyl cellulose (CMC), polyvinylpyrrolidone (PVP), and sodium dodecyl sulfate (SDS) were used to prepare modified nZVI. The modifiers increased electrostatic repulsion and steric hindrance, inhibited aggregation, and maintained reactivity. The modified particles were smaller (5–15 nm vs. ~50 nm for nZVI) and more dispersible. In three heavy metal-contaminated soils from Zn–Pb mine areas in China, modified nZVI showed improved mobility and retention; column effluent Fe reached 595.5 mg/L for P-nZVI in S1 and 639.6 mg/L for C-nZVI in S3, although mobility varied with soil properties. After stabilization, TCLP leaching of Zn, Cr, Pb, and Cu decreased significantly. Zn leaching was reduced by up to 95.0% (P-nZVI, S3) and 93.49% (C-nZVI, S2); Cr leaching was reduced by up to 98.6% (S-nZVI, S3) and 97.0% (P-nZVI, S3); Pb and Cu reached below detection limits under optimized treatments (C-nZVI or P-nZVI at 1.0 g/L Fe) in selected soils. The K value, defined as (F1 + F2)/(F3 + F4 + F5), decreased by up to 16.25% for Zn, 11.88% for Cr, 13.63% for Cu, and 10.04% for Pb. Because K represents the ratio of labile to stable fractions, its decrease indicates a shift from labile to stable fractions, enhanced immobilization, and reduced bioavailability. Overall, PVP-modified nZVI performed best for Zn and Cr, whereas CMC-modified nZVI was most suitable for Pb and Cu.

## 1. Introduction

Human activities such as mining, smelting, irrigation, sludge application, and fertilizer/pesticide use have caused widespread soil heavy metal pollution, threatening ecological security, food safety, and human health [1,2,3]. In China, heavy metals account for more than 80% of soil pollutants in contaminated cropland, mainly due to mining and metallurgical activities [4,5]. Mine-impacted soils are often co-contaminated by multiple metals such as Cd, Hg, As, Cr, Pb, Zn, and Cu, and their interactions complicate remediation [3,6]. Because heavy metals are non-degradable, stabilization—reducing their mobility and bioavailability—is a cost-effective and environmentally friendly remediation strategy [4,7].

The non-degradable nature of heavy metals determines their long-term persistence in soil [4]. Stabilization, which reduces the bioavailability and mobility of heavy metal pollutants in the soil profile, is a cost-effective and environmentally friendly remediation approach that has gained considerable attention [7]. Zero-valent iron technology, owing to its simple operation, high treatment efficiency, low operating cost, low energy consumption, and abundant material supply, is considered a promising soil stabilization technology. In particular, nano zero-valent iron (nZVI) possesses reduction and adsorption capacities that differ considerably from those of conventional millimeter- or micrometer-scale zero-valent iron, and can efficiently transform environmental pollutants such as chlorinated organics, toxic metals, and inorganic compounds into less toxic or inert substances [8,9,10,11,12,13]. For example, field trials conducted by the US EPA at more than 40 trichloroethylene (TCE)/dense non-aqueous phase liquid (DNAPL)-contaminated groundwater sites using iron-based nanotechnology have achieved contaminant removal rates of more than 98% within short treatment periods [14]. In addition to the remediation of polluted soils, nZVI has also been applied to the treatment of water bodies contaminated with heavy metals such as Cd, As, Cr, Zn, Ni, Ag, and Pb [3,15].

However, electrostatic attraction between particle surfaces, high surface energy, van der Waals attraction, and hydrogen bonding and other chemical bonds on particle surfaces facilitate the formation of nanoparticle aggregates; moreover, nZVI particles are prone to oxidation. These factors restrict the dispersibility and mobility of nZVI in contaminated water and soil, seriously limiting its remediation capacity [2]. To overcome these shortcomings, stabilizers such as polyelectrolytes, surfactants, and polysaccharides, as well as surface metal loading, activated carbon/biochar loading, and emulsified carriers, have been used to surface-modify nZVI [16,17,18,19]. Among the various stabilizers, surfactants are widely used because of their ability to keep particles colloidally dispersed, whereas the water-soluble polysaccharide/polyelectrolyte carboxymethyl cellulose (CMC) and water-soluble starch are considered environmentally friendly and economical stabilizers [4,17]. Stabilizer-modified nZVI exhibits good stability, mobility, and reactivity in soil and groundwater and can readily reach contaminated subsurface zones with high remediation efficiency; when injected in situ into soil, it can reach groundwater pollution sources and achieve satisfactory remediation of organic pollutants and heavy metals [2,4,17].

Because stabilizers differ in type and properties, the properties of the resulting nanoparticles also differ. For example, the specific surface area of cetylpyridinium chloride-modified nZVI determined by the Brunauer–Emmett–Teller (BET) nitrogen adsorption method was 25.4 m^2^/g [20], whereas that of unmodified nZVI was approximately 55 m^2^/g [21]. Ponder et al. [22] found that PolyFlo resin-modified nZVI had a specific surface area of 24.4 ± 1.5 m^2^/g, which was higher than that of unmodified nZVI (21.7 ± 1.5 m^2^/g). Numerous studies have addressed modified nZVI for the remediation of waters polluted with heavy metals or organic compounds, whereas fewer have focused on soils, in which dissolved organic matter (DOM), pH, Cl^−^, HCO_3_^−^, PO_4_^3−^, Ca^2+^, humic acid, and other factors can also affect heavy metal removal by iron nanoparticles [2,4,23,24].

Modified nZVI has been used as a soil stabilizer for sites contaminated with single heavy metals such as As, Cr(VI), and Hg, with good stabilization effects [2,4,25,26,27]. However, studies on soils contaminated by multiple metals remain scarce [28,29]. The mechanisms of heavy metal removal by nZVI have mostly been studied in water and include (1) reduction of metals with standard redox potentials higher than that of Fe^2+^/Fe (−0.41 V), (2) adsorption onto small particles with large specific surface areas and high surface energy, and (3) co-precipitation with OH^−^ generated from Fe^0^–water reactions [2,4]. In soil, heterogeneity, particle-size fractions, pH, cation exchange capacity (CEC), organic matter, and competitive or synergistic interactions among coexisting metals can further influence stabilization [2,30,31,32]. Recent reviews of carbon- and biochar-supported nZVI highlight their promise for heavy metal remediation [33,34], but soil-specific mechanistic studies remain limited.

In this study, nZVI was modified with stabilizers of different properties: SDS (anionic surfactant), PVP (non-ionic polymer), and CMC (anionic polyelectrolyte/soluble biopolysaccharide). The modified nZVIs were applied to three soils from typical zinc–lead mine areas in China, contaminated with Zn, Cr, Pb, and Cu and differing in physicochemical properties. The specific objectives were to (1) evaluate the self-stability of different modified nZVIs and their stability and mobility in soil; (2) compare the stabilization efficiency of different modified nZVIs for heavy metals in contaminated soils; and (3) explore the mechanisms underlying heavy metal stabilization by modified nZVIs in soil.

Although the synthesis and characterization protocols used in this study were adapted from established methods [19,25], this study provides new insights by systematically comparing three stabilizer-modified nZVIs (S-nZVI, C-nZVI, and P-nZVI) in three real multi-metal-contaminated soils from contrasting Zn–Pb mining regions of China. Unlike most previous studies that focused on single-metal systems or aqueous media, this work (i) links soil physicochemical properties to the mobility and retention of modified nZVI, (ii) identifies stabilizer-specific selectivity for Zn, Cr, Pb, and Cu, and (iii) quantifies the redistribution of heavy metals among geochemical fractions and the associated decrease in K values.

## 2. Materials and Methods

### 2.1. Study Sites and Soil Sampling

Sample 1 (S1) was collected from the Fankou lead—zinc mine, Renhua County, Shaoguan City, Guangdong Province, southeastern China (Figure 1). The Fankou lead–zinc mine was established in 1958 and has been in operation since 1968; it is one of the major lead–zinc producers in China. Sample 2 (S2) was collected from the Fuyang Jinxin mine in Fuyang District, Hangzhou City, Zhejiang Province, eastern China, which was established in 1997 (Figure 1). Sample 3 (S3) was collected from the Lijiaqou lead–zinc deposit, Longnan City, Gansu Province, northwestern China. All soil samples were loamy-sand and granular-sandy soils. Sampling was conducted at a depth of 0–20 cm. This depth represents the typical plow layer zone, where heavy metals tend to accumulate and where ecological risks and human exposure are most relevant; it is also the standard sampling depth for soil contamination assessment under the Specification of Land Quality Geochemical Assessment (DZ/T 0295-2016) [35]. At each site, five subsamples were collected per plot and mixed to obtain a composite sample, which was placed in clean bags, dried indoors, and sieved (<10 mesh). Sampling was carried out in [month/year] during the dry season. The weather during sampling was sunny, with daily air temperatures of 15–22 °C. If the three sites were sampled at different times, the sampling time and weather for each site were as follows: S1: [month/year, season, weather]; S2: [month/year, season, weather]; S3: [month/year, season, weather]. Soil pH, soil organic matter (SOM), CEC, and particle-size distribution were determined following standard methods [36].

### 2.2. Preparation of Modified nZVI Particles

The nZVIs were prepared by the liquid-phase reduction method. An aqueous solution of 0.026 M NaBH_4_ was slowly added dropwise to a 0.012 M FeCl_3_·6H_2_O aqueous solution at 25 °C under atmospheric pressure with electric stirring. The generated iron nanoparticles were filtered using a vacuum pump and washed with 1 M HCl to remove surface oxides and other contaminants. After acid washing, the iron particles were washed again with oxygen-free distilled water and anhydrous ethanol, filtered, and dried under vacuum. The dried modified nZVIs were stored under vacuum in sealed brown containers at room temperature and used within [X] days to minimize oxidation. All suspensions were prepared freshly before each experiment. No inert gas (N_2_ or Ar) was used during synthesis; therefore, partial surface oxidation cannot be completely excluded, but the stabilizer coating and vacuum storage were used to limit oxidation. Subsequently, SDS was added to the nZVI suspension at its critical micelle concentration (0.0082 M) to form SDS-modified nZVI (S-nZVI) [25]. CMC-modified nZVI (C-nZVI) was prepared using 0.2 wt.% CMC following the method of He and Zhao [37]. PVP-modified nZVI (P-nZVI) was prepared by adding 0.2 wt.% PVP-K30 as a stabilizer [19].

### 2.3. Characterization of Modified nZVI

The morphology of the three modified nZVIs was characterized before and after modification by high-resolution transmission electron microscopy (HR-TEM) on freeze-dried samples and by field emission scanning electron microscopy (FE-SEM). The specific surface area was determined by the BET nitrogen adsorption method using a fully automated specific surface area and pore-size distribution analyzer. The surface structure and composition of the modified nZVIs were characterized by X-ray diffraction (XRD). The zeta potential was measured to characterize the stability of the colloidal dispersion systems [18].

### 2.4. Column Mobility Experiments of nZVIs in Soil

Air-dried, ground soil (9 g) was passed through a 2-mm sieve and packed into a Plexiglas column with an inner diameter of 1.0 cm and a length of 10 cm. The soil was packed in small increments with gentle tapping to a bed height of approximately 6.3 cm. The column volume was 7.85 cm^3^. The bulk density was approximately 1.15 g/cm^3^. Assuming a particle density of 2.65 g/cm^3^, the porosity was calculated as 0.566, and the pore volume was approximately 4.44 mL. The column was pre-equilibrated with deionized water before injection.

Subsequently, suspensions of nZVI and the three modified nZVIs (S-nZVI, C-nZVI, and P-nZVI; 1.0 g/L Fe) were injected from the top of the column at a rate of 0.18 mL/min using a peristaltic pump. The corresponding Darcy velocity was 0.229 cm/min, and the pore-water velocity was approximately 0.405 cm/min. The effluent was collected at 10, 20, 30, 40, 50, and 60 min, filtered through a 0.22-μm filter membrane, and acidified with 0.1 M HCl. The injection period was divided into three stages: Stage I (0–20 min), Stage II (20–40 min), and Stage III (40–60 min). The Fe content was determined by AAS or ICP-MS, using pure water as the blank. The experiment was performed in triplicate [16].

### 2.5. Batch Retention Experiments of nZVIs in Soil

A 12-g aliquot of sieved soil was added to a 100-mL centrifuge tube with 63 mL of deionized water and allowed to stand at room temperature for 14 days. Subsequently, 93% of the supernatant was replaced with the same volume of nZVI, S-nZVI, C-nZVI, or P-nZVI suspension (Fe = 0.3 g/L), and the tubes were placed on a shaker. At 10, 20, 30, 40, 50, and 60 h, the supernatant was centrifuged at 400× *g* for 10 min to separate free nZVIs from the soil. The Fe content was determined by AAS or ICP-MS, and the amount of modified nZVIs retained by the solid phase was obtained by calculation. Deionized water was used as a blank instead of soil, and the experiment was performed in triplicate [13].

### 2.6. Heavy Metal Stabilization Experiments

Soil samples were treated with nZVI, C-nZVI, P-nZVI, and S-nZVI at doses of 0.5 and 1.0 g/L Fe and incubated for 14 days at room temperature, with untreated soil as the control. Heavy metal leaching toxicity was then determined using the toxicity characteristic leaching procedure (TCLP) of USEPA Method 1311 [38], and the leachate concentrations were compared with the regulatory limits for hazardous waste identification (GB 5085.3-2007) [39]. The chemical speciation of Zn, Cr, Pb, and Cu was determined using a five-step sequential extraction procedure [40], which partitions metals into exchangeable (F1), carbonate-bound (F2), Fe/Mn oxide-bound (F3), organic matter-bound (F4), and residual (F5) fractions. The stability coefficient K, defined as K = (F1 + F2)/(F3 + F4 + F5), was used to evaluate the stabilization effect; a lower K value indicates a greater proportion of stable metal fractions. Metal ion concentrations were determined by AAS and ICP-MS. The experiment was performed in triplicate. TCLP was performed according to USEPA Method 1311. Extraction fluid [X] was used, which was prepared with [5.7 mL glacial acetic acid and 64.3 mL 1 M NaOH diluted to 1 L, pH 4.93 ± 0.05] or [5.7 mL glacial acetic acid diluted to 1 L, pH 2.88 ± 0.05]. The leachate pH was recorded before analysis.

### 2.7. Leaching Column Experiments of Stabilized Soils

Batch leaching experiments were performed according to Chrysochoou et al. [41]. Briefly, 9 g of soil stabilized by nZVI, S-nZVI, P-nZVI, or C-nZVI for 14 days was packed into a Plexiglas column with a diameter of 1.0 cm and a height of 10 cm and leached with simulated groundwater [42]. The leachate was collected at 0, 75, and 350 h and passed through a 0.22-μm filter membrane for the determination of pH, Eh, heavy metal concentrations, and Fe content. Soils without nZVIs were used as blanks, and the experiment was performed in triplicate.

### 2.8. Statistical Analysis

Graphs were generated using Origin software (10.3, OriginLab Corporation, Northampton, MA, USA). Statistical analyses were performed using Microsoft Excel. Differences among treatments were evaluated using the Friedman test (*p* < 0.05). Pearson correlation analysis was used to relate the Fe retention of the nZVIs to soil properties.

## 3. Results and Discussion

### 3.1. Characterization, Mobility, and Retention of Modified nZVIs

#### 3.1.1. TEM Characterization

The transmission electron micrographs (TEM) of the four experimentally prepared nZVIs are shown in Figure 2. There were considerable differences in the morphology of nZVIs before and after modification. As shown in Figure 2a, the unmodified nZVIs had a particle size of about 50 nm, were spherical in shape, and showed an overall black, agglomerated, chain-like structure. In general, nZVIs readily agglomerate through the combined effects of magnetic interactions, van der Waals forces, and surface tension. Because the particle size of nZVIs is smaller than the wavelength of visible light, they absorb light waves and approach an ideal black body. As shown in Figure 2b, owing to the presence of CMC, which effectively overcomes the magnetic interactions, the C-nZVIs were less prone to agglomeration under the effects of electrostatic repulsion and steric hindrance and presented a highly dispersed state, with a reduced particle size of about 5 nm. Figure 2c shows that the P-nZVI particles were somewhat aggregated, mostly because of magnetic interactions among the nZVI particles. The prepared P-nZVI particles had a particle size of about 15 nm and an outer coating film, preventing their oxidation. Figure 2d shows that the prepared S-nZVIs were well dispersed, with a particle size of approximately 8 nm. According to the TEM characterization, the nZVI particles modified by CMC, PVP, and SDS exhibited uniform particle distribution and good dispersion without agglomeration, with substantially smaller particle sizes than unmodified nZVI particles.

These results provide new evidence that CMC, PVP, and SDS modification can reduce nZVI particle size from ~50 nm to 5–15 nm and improve dispersion in real soil remediation scenarios.

#### 3.1.2. Column Mobility Experiments

The results of the mobility experiments are shown in Table 1. The column was packed with 9 g of 2-mm sieved soil to a bed height of approximately 10 cm. The column volume was 7.85 cm^3^, the bulk density was approximately 1.15 g/cm^3^, the porosity was approximately 0.566, and the pore volume was approximately 4.44 mL. The flow rate was 0.18 mL/min, and the effluent was collected over three stages: Stage I (0–20 min), Stage II (20–40 min), and Stage III (40–60 min).

For the highly contaminated soil S1 from Renhua, Guangdong Province, the nZVI, C-nZVI, and S-nZVI treatments differed significantly from the control, and the Fe content in the effluent decreased with injection time, indicating that the Fe nanoparticles in these treatments were retained by S1 as injection proceeded and that their mobility was poorer. In contrast, P-nZVI showed the highest mobility in this soil, with Fe concentrations in the effluent increasing from 152.7 mg/L (stage I) to 295.2 mg/L (stage II) and 595.5 mg/L (stage III), significantly different from the other treatments. This can be explained by the acidic pH of S1: as PVP is a non-ionic polymer with strong hydrophilic properties, P-nZVI has no electrostatic interaction with the charged surfaces in soil S1, and its migration ability was therefore higher. According to previous studies, SDS solutions and foams effectively promote the migration of nZVI in porous media, as shown in batch and simulated column experiments [3,4]. However, because SDS is an anionic surfactant, S-nZVI does not easily migrate in S1, as it reacts electrostatically with H^+^ in the soil under acidic conditions. In addition, under acidic conditions, the carboxyl groups of CMC are prevented from ionizing, resulting in a decrease in viscosity [30], which in turn decreases the migration capacity of C-nZVI, which is prone to particle surface oxidation and agglomeration in soil solutions.

For the highly contaminated soil S2 from Fuyang District, Hangzhou, Zhejiang Province, nZVI, C-nZVI, P-nZVI, and S-nZVI showed poor migration ability, and C-nZVI and P-nZVI were gradually retained over time. The Fe content in the effluent decreased sharply at stages II and III, reaching values below the detection limit, whereas S-nZVI gradually migrated owing to the saturation of the soil adsorption capacity. This can be explained by the high content of soil organic matter and the high cation exchange capacity of S2, which result in a strong retention capacity for nZVI before and after modification and, consequently, a decreased migration capacity. In related studies, the migration capacity of C-nZVI was enhanced when the particle size of the filling medium was changed from 20–30 mesh to 10–20 mesh quartz sand: as the particle size decreases, the specific surface area increases, the retention capacity of the packed medium for nZVI increases, and free diffusion is weakened, which eventually leads to substantial deposition of modified and unmodified nZVI on the surface of the porous medium [32]. The CMC adsorbed on the nZVI surface and free CMC can provide steric stabilization and depletion stabilization, respectively, forming a stable C-nZVI suspension [41]. In addition, continuous experiments have demonstrated the good mobility of CMC-stabilized Fe^0^ in actual groundwater and soil [43]. The feasibility of Cr(VI) treatment in groundwater and sandy soils has been investigated in intermittent and continuous experiments [26]. Based on the results, CMC-stabilized nZVI showed greater activity and reaction rates along with good mobility in soil; Cr(VI) was converted into the less toxic Cr(III) [26,44], with a higher reactivity in the pH range of 5.0–9.0. The CMC modified the surface of the nZVI particles, increasing the steric hindrance and electrostatic repulsion between particles, which raised the potential barrier and thus formed a more kinetically stable C-nZVI, greatly reducing its agglomeration and providing it with stronger migration ability in soil.

Overall, the results of this experiment showed that the modified nZVIs migrated relatively well in the three soils compared with unmodified nZVI, mainly because nZVI was prone to agglomeration, impeding soil migration, whereas the modifiers CMC, PVP, and SDS effectively increased the steric hindrance and electrostatic repulsion on the Fe^0^ surface and protected Fe^0^ from oxidation and agglomeration, thereby improving nZVI stability and mobility in soil. Different modified nZVIs showed different migration abilities in soils with different properties. In actual soil remediation projects, small-scale and pilot tests are needed to screen the most mobile modified nZVIs.

A key new finding is that the mobility of modified nZVI was soil-specific: P-nZVI showed the highest mobility in acidic S1, whereas C-nZVI showed the highest mobility in S3, indicating that stabilizer selection should be tailored to soil properties.

#### 3.1.3. Retention and Adsorption Kinetics

As shown in Figure 3a, for the four treatments, the fitted maximum Fe retention capacities (ymax) obtained for soil S1 with the pseudo-second-order kinetic adsorption model were 262.64, 271.03, 227.10, and 264.20 mg/kg, respectively, which were closer to the experimental results than the values obtained with the pseudo-first-order kinetic model (141.46, 107.55, 62.74, and 170.20 mg/kg, respectively). Comparison of the model fit correlation coefficients (R^2^) indicated that the kinetic adsorption data of S1 for Fe under the four treatments were more consistent with the pseudo-second-order kinetic adsorption model; that is, the adsorption rate was higher at the beginning of adsorption and slowed to a certain value as adsorption equilibrium was reached.

As shown in Figure 3b, for the four treatments, the ymax values obtained for soil S2 with the pseudo-second-order kinetic model were 253.49, 213.90, 254.75, and 244.36 mg/kg, respectively, closer to the experimental results than the pseudo-first-order values (141.46, 107.55, 62.74, and 170.20 mg/kg, respectively). The comparison of R^2^ showed that the kinetic adsorption data of S2 for Fe under the four treatments were more consistent with the pseudo-second-order kinetic model, similar to the results obtained for S1.

As shown in Figure 3c, for the four treatments, the ymax values of Fe retention by soil S3 obtained with the pseudo-second-order kinetic model were 253.52, 261.17, 254.09, and 231.61 mg/kg, respectively, closer to the experimental results than the pseudo-first-order values (141.46, 107.55, 62.74, and 170.20 mg/kg, respectively). The comparison of R^2^ again showed that the kinetic adsorption data of S3 were more consistent with the pseudo-second-order kinetic model, similar to those for S1. Zhang et al. [4] investigated the feasibility of nZVI treatment in actual groundwater and sandy soils through intermittent and continuous experiments. The results showed that nZVIs stabilized by CMC exhibited greater activity and reaction rates than ordinary particles, and that 0.08 g/L of C-nZVI could rapidly reduce 34 mg/L of Cr(VI), with an initial pseudo-first-order reaction rate of 0.08 h^−1^. When the nZVI concentration was increased from 0.04 to 0.12 g/L, the Cr(VI) removal efficiency increased from 24% to 90%. More recently, CMC-coated nZVI has also been shown to effectively reduce Cr(VI) in contaminated soils [45].

To analyze the influence of soil properties on Fe retention, Pearson correlation analysis was performed to relate the retention amount in each treatment to soil texture, pH, organic matter, and cation content (Table 2). Based on the results, SOM, pH, and the sand, silt, and clay contents had the greatest impacts on soil retention of unmodified nZVI, and the sand content was negatively correlated with retention intensity. For C-nZVI, pH, organic matter content, and clay content mostly affected nZVI retention. Similar studies have shown that as the medium particle size decreases and the specific surface area increases, the retention capacity of the packed medium for nZVIs increases and free diffusion is weakened, eventually leading to the massive deposition of modified and unmodified nZVIs on the surface of the porous medium [3]. For P-nZVI, the effect of pH was relatively weak because PVP is a non-ionic polymer; in contrast, SOM and clay particle content had the highest impact on nZVI retention. For S-nZVI, soil pH and clay particle content were the most important factors affecting soil retention. As previously shown [9], soils with high SOM content and CEC have a strong retention capacity for heavy metals, which is consistent with the results of the present study. It should be noted that only three soils were examined; therefore, the correlation results should be interpreted with caution as an exploratory analysis.

#### 3.1.4. Role of Stabilizer Capping in Mobility and Adsorption

The capping layer controls both the transport of nZVI in soil and its adsorption/reaction with heavy metals. CMC is an anionic polyelectrolyte. Its carboxyl groups anchor onto the nZVI surface, while the long hydrophilic chains extend into solution, providing steric hindrance and, at circumneutral to alkaline pH, strong electrostatic repulsion. This explains the high mobility of C-nZVI in S3, where effluent Fe reached 639.6 mg/L. In acidic S1, however, partial protonation of carboxyl groups reduces the negative charge and viscosity, leading to aggregation and retention. The carboxyl groups can also complex Pb(II) and Cu(II), which is consistent with the best stabilization of Pb and Cu by C-nZVI.

PVP is a non-ionic polymer. Its carbonyl groups coordinate with surface Fe and hydrogen-bond with surface hydroxyls, forming a protective film. Because PVP carries no charge, P-nZVI experiences weak electrostatic interaction with soil surfaces, explaining its highest mobility in acidic S1, where effluent Fe reached 595.5 mg/L. The PVP film also limits oxidation and preserves Fe^0^ reactivity, favoring Cr(VI) reduction and Zn stabilization.

SDS is an anionic surfactant. It adsorbs with its anionic head groups oriented outward, forming hemimicelles or a bilayer, which increases negative surface charge and dispersion. In acidic soils, the increased positive charge on soil surfaces enhances electrostatic attraction with anionic SDS, reducing S-nZVI mobility. In soils with high clay and SOM content, such as S2, deposition and retention are further enhanced. SDS also improves dispersion and exposes more Fe^0^ sites for Cr(VI) reduction, although it may compete with anionic metal species for surface sites.

The pseudo-second-order adsorption kinetics indicate that chemisorption-like interactions dominate Fe retention. The capping layer affects the accessibility of surface sites and the affinity for metal ions. Correlation analysis (Table 2) further shows that pH, SOM, and clay content are key factors controlling Fe retention, with the effect of pH being weakest for non-ionic PVP. Overall, the capping characteristics—charge, functional groups, hydrophilicity, and steric bulk—determine the trade-off between mobility and adsorption capacity, and no single stabilizer is optimal for all soils and metals.

### 3.2. Stabilization Efficiency of Different nZVIs for Heavy Metal-Contaminated Soils

#### 3.2.1. Leaching Toxicity of Heavy Metals in Soil S1 After Stabilization

The TCLP-extracted Zn, Cr, and Pb contents in soil S1 exceeded the regulatory threshold values, indicating considerable ecological risks. Leaching of the four heavy metals in soil S1 was significantly reduced by 0.5 and 1.0 g/L Fe of nZVI, C-nZVI, P-nZVI, and S-nZVI compared with the control. As shown in Figure 4, the leaching toxicity of Zn in the soil was most significantly reduced by the P-nZVI treatment, with a 66.48% to 87.95% reduction compared with the control, followed by S-nZVI, C-nZVI, and nZVI, whose TCLP extracts reduced the leaching toxicity by 59.4% to 78.61%, 42.67% to 58.1%, and 41.11% to 46.6%, respectively. This indicates that the stability of Zn was improved; the most suitable treatment was the application of P-nZVI at a dose of 1.0 g/L Fe, followed by S-nZVI at 1.0 g/L Fe. Regarding Cr, Figure 4b shows that S-nZVI and P-nZVI were more effective than nZVI and C-nZVI, whereas for Pb, C-nZVI and P-nZVI at a dose of 1.0 g/L Fe were most suitable. The leaching toxicity of Cu was below the detection limit after treatment with P-nZVI at a dose of 1.0 g/L Fe, which was the most suitable approach.

#### 3.2.2. Leaching Toxicity of Heavy Metals in Soil S2 After Stabilization

The TCLP-extracted Zn, Cr, and Pb contents in soil S2 exceeded the regulatory threshold values, indicating considerable ecological risks. At doses of 0.5 and 1.0 g/L Fe of nZVI, C-nZVI, P-nZVI, and S-nZVI, leaching of the four heavy metals was considerably reduced compared with the control. As shown in Figure 5, the leaching toxicity of Zn was most significantly reduced by the C-nZVI treatment, with a 61.43% to 93.49% reduction compared with the control, followed by stabilization with S-nZVI, P-nZVI, and nZVI, with reductions of 55.14% to 78.51%, 29.28% to 57.39%, and 11.66% to 20.37%, respectively. This indicates that the stability of Zn in the soil was significantly improved, with the best treatment effect obtained with C-nZVI at 1.0 g/L Fe, followed by S-nZVI at 1.0 g/L Fe. According to Figure 5, compared with nZVI and P-nZVI, C-nZVI and S-nZVI were more effective in treating Cr, whereas for Pb, C-nZVI and S-nZVI at a dose of 1.0 g/L Fe obtained the best results. The highest stabilization of Cu was achieved using C-nZVI at a dose of 1.0 g/L Fe.

#### 3.2.3. Leaching Toxicity of Heavy Metals in Soil S3 After Stabilization

The leaching toxicity of Zn in the soil was most significantly reduced by the P-nZVI treatment, with an 80.2% to 95% reduction compared with the control, followed by S-nZVI, C-nZVI, and nZVI, with reductions of 56.0% to 81.1%, 40.7% to 69.2%, and 13.5% to 44.7%, respectively. The stability of Zn was significantly higher after stabilization, with the best treatment effect of P-nZVI at a dose of 1.0 g/L Fe, followed by S-nZVI at 1.0 g/L Fe and P-nZVI at 0.5 g/L Fe. The leaching content of Zn in the soil under all treatments was below the regulatory threshold.

The leaching toxicity of Cr and Pb in the soil was significantly reduced after stabilization with nZVI, C-nZVI, P-nZVI, and S-nZVI at 0.5 and 1.0 g/L Fe compared with the control and was below the regulatory threshold. This indicates that nZVI, before and after modification, can effectively stabilize Cr and Pb in soil, thereby reducing the ecological risk. As shown in Figure 6, S-nZVI and P-nZVI were more effective in stabilizing Cr than nZVI and C-nZVI, resulting in leaching toxicity reductions of 98.6% and 97.0%, respectively. For Pb, C-nZVI and P-nZVI at a dose of 1.0 g/L Fe were most effective and resulted in Pb levels below the detection limit.

The leaching toxicity of Cu in the soil was not significantly different after treatment with 0.5 g/L Fe nZVI compared with the control, and the best results were obtained with C-nZVI at a dose of 1.0 g/L Fe, decreasing the leaching toxicity to levels below the detection limit.

Importantly, the stabilization efficiency was metal-specific: PVP-nZVI performed best for Zn and Cr, whereas CMC-nZVI was most suitable for Pb and Cu. This selectivity has not been systematically reported for these three stabilizers in multi-metal-contaminated soils.

#### 3.2.4. Discussion of the Stabilization Efficiency

The TCLP results indicate that the stabilization efficiency was both metal-specific and soil-specific. In S1, P-nZVI was most effective for Zn and Cu, whereas S-nZVI and P-nZVI performed best for Cr, and C-nZVI and P-nZVI were most suitable for Pb. The strong performance of P-nZVI can be attributed to the non-ionic PVP coating, which limits nZVI oxidation and preserves Fe^0^ reactivity, thereby maintaining reduction and adsorption sites. For Cr(VI), reduction to Cr(III) followed by precipitation as Cr(OH)_3_ or Cr_x_Fe_1−x_(OH)_3_ is likely the dominant mechanism, consistent with the high Cr stabilization by S-nZVI and P-nZVI. For Pb and Cu, CMC provides carboxyl groups that can complex these divalent metals and enhance their retention, explaining why C-nZVI was most effective for Pb and Cu in S1 and S2.

Soil properties further modulated the stabilization performance. In S2, the high SOM and CEC increased nZVI retention and may also provide additional sorption sites; however, strong nZVI retention did not always translate into the highest stabilization efficiency, indicating that metal–stabilizer complexation and competition with soil organic matter also control the outcome. In S3, P-nZVI was best for Zn, while C-nZVI was best for Cr, Cu, and Pb, suggesting that soil pH and mineral composition influence the dominant stabilization mechanism. The dose effect was also evident: 1.0 g/L Fe generally outperformed 0.5 g/L Fe because of the greater number of reactive sites, although the improvement was not always linear, likely due to particle aggregation or surface saturation.

Overall, these results demonstrate that no single modified nZVI is optimal for all metals. Stabilizer selection should consider both the target metal and soil physicochemical properties. The correlation analysis in Table 2 supports the important role of pH, SOM, and clay content in Fe retention, although the small number of soils means these correlations should be regarded as exploratory.

### 3.3. Changes in Heavy Metal Speciation and Stability After Stabilization

After stabilization by nZVI, C-nZVI, S-nZVI, and P-nZVI, the heavy metals (Cr, Cu, Zn, and Pb) in S1, S2, and S3 all shifted from labile fractions (exchangeable and carbonate-bound) to stable fractions (Fe/Mn oxide-bound, organic matter-bound, and residual), and the proportion of stable fractions increased significantly. Consequently, the K values decreased, indicating reduced bioavailability of Cr, Cu, Zn, and Pb.

As shown in Figure 7a, the K value of Cr in soil S1 changed most significantly with the addition of P-nZVI, decreasing by 10.27%, followed by treatment with C-nZVI (6.55%), nZVI (4.96%), and S-nZVI (4.81%). In addition, the K value of Cu in the soil changed most significantly after P-nZVI remediation, decreasing by 6.23%, followed by C-nZVI (4.32%), S-nZVI (3.93%), and nZVI (2.45%) (Figure 7b). Also, the K value of Zn changed most significantly after the addition of P-nZVI, decreasing by 15.63%, followed by S-nZVI (8.87%), C-nZVI (3.79%), and nZVI (2.93%) (Figure 7c). However, the K value of Pb in the soil changed most significantly after the addition of C-nZVI, decreasing by 9.93%, followed by P-nZVI (7.89%), nZVI (5.50%), and S-nZVI (5.20%) (Figure 7d). These results indicate that P-nZVI was most effective in stabilizing Cr, Cu, and Zn, whereas C-nZVI most effectively stabilized Pb in soil S1.

As shown in Figure 8a, the K value of Cr in soil S2 changed most significantly after the addition of P-nZVI, with a reduction of 8.50%, followed by S-nZVI (7.16%) and C-nZVI (2.23%). Based on this, P-nZVI most effectively stabilized Cr in soil S2. In addition, the K value of Cu in the soil changed most significantly with P-nZVI remediation, decreasing by 6.75%, followed by S-nZVI (6.46%), C-nZVI (3.52%), and nZVI (3.13%) (Figure 8b). Based on these findings, P-nZVI was most effective in stabilizing Cu in soil S2. Also, the K value of Zn in the soil changed most significantly with P-nZVI remediation, decreasing by 14.41%, followed by S-nZVI (7.16%), nZVI (6.21%), and C-nZVI (2.40%) (Figure 8c). This indicates that P-nZVI most effectively stabilized Zn in soil S2. The K value of Pb in the soil changed most significantly after P-nZVI remediation, decreasing by 6.53%, followed by nZVI (4.60%), C-nZVI (3.52%), and S-nZVI (1.80%) (Figure 8d). Based on these findings, P-nZVI was most effective in stabilizing Pb in soil S2.

According to Figure 9a, the K value of Cr in soil S3 changed most significantly after the addition of C-nZVI, with a reduction of 11.88%, followed by S-nZVI (7.12%), P-nZVI (6.62%), and nZVI (5.02%). Accordingly, C-nZVI most effectively stabilized Cr in soil S3. In addition, the K value of Cu in soil S3 changed most significantly with the addition of C-nZVI, decreasing by 13.63%, followed by P-nZVI (10.94%), S-nZVI (9.22%), and nZVI (6.67%) (Figure 9b). Accordingly, C-nZVI was most effective in stabilizing Cu in soil S3. Also, the K value of Zn in soil S3 changed most significantly with the addition of P-nZVI, decreasing by 16.25%, followed by nZVI (7.22%) and S-nZVI (5.83%) (Figure 9c). Based on this, P-nZVI was most effective in stabilizing Zn in soil S3. However, the K value of Pb in soil S3 changed most significantly after the addition of C-nZVI, decreasing by 10.04%, followed by nZVI (5.00%), S-nZVI (4.63%), and P-nZVI (3.98%) (Figure 9d). This suggests that C-nZVI was most effective in stabilizing Pb in soil S3.

These results quantify a new finding: after stabilization, all four heavy metals shifted from labile to stable fractions, with maximum K-value reductions of 16.25% for Zn, 11.88% for Cr, 13.63% for Cu, and 10.04% for Pb.

## 4. Conclusions

The use of CMC, PVP, and SDS as dispersants and stabilizers to prepare nZVIs increased the electrostatic repulsion and steric hindrance between particles, which effectively inhibited particle agglomeration and enhanced nZVI dispersion, thereby maintaining the high reactivity of the particles. The prepared iron nanoparticles were smaller in size, had higher specific surface areas, and showed improved adsorption capacity. The modified nZVIs showed good migration and stabilization ability in soil. For different soils, the mobility and stability of different nZVIs differed with the physicochemical properties and pore-size distribution of the soils. After stabilization with the three modified nZVIs, the leaching toxicity of heavy metals in the soil was reduced, thereby decreasing the ecological risk. For Zn and Cr, PVP-modified nZVI achieved the best treatment effect, whereas for Pb and Cu, CMC-modified nZVI was the most suitable. After stabilization by nZVI, C-nZVI, S-nZVI, and P-nZVI, the four heavy metals in the S1, S2, and S3 soils shifted to more stable fractions, resulting in lower K values and decreased bioavailability. Because the synthesis was performed under atmospheric conditions without inert gas protection, partial surface oxidation of nZVI cannot be excluded. Future studies should use N_2_/Ar-purged or glovebox-based synthesis to further improve Fe^0^ stability.

## Figures and Tables

**Figure 1:**
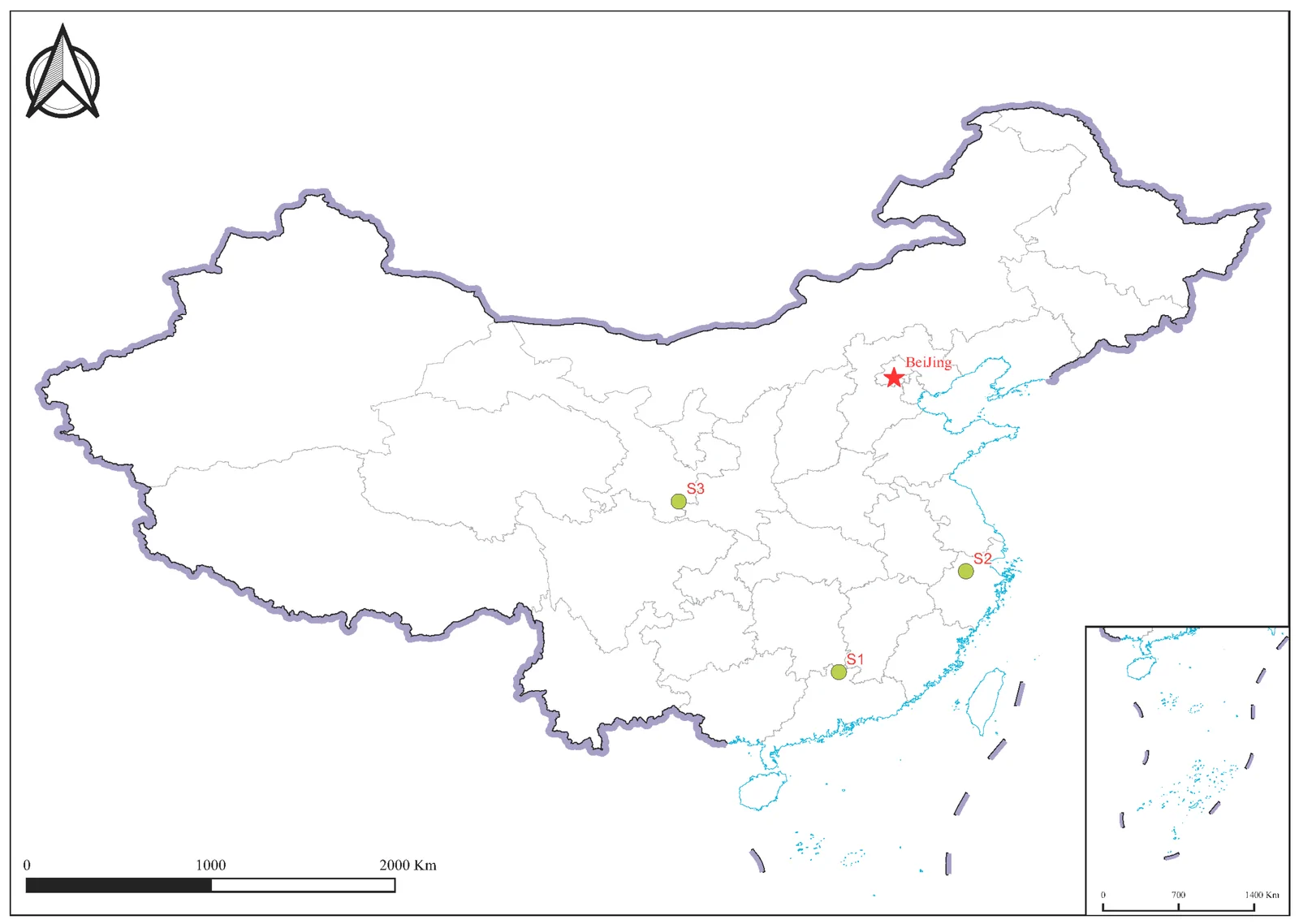
Locations of the sampling sites.

**Figure 2:**
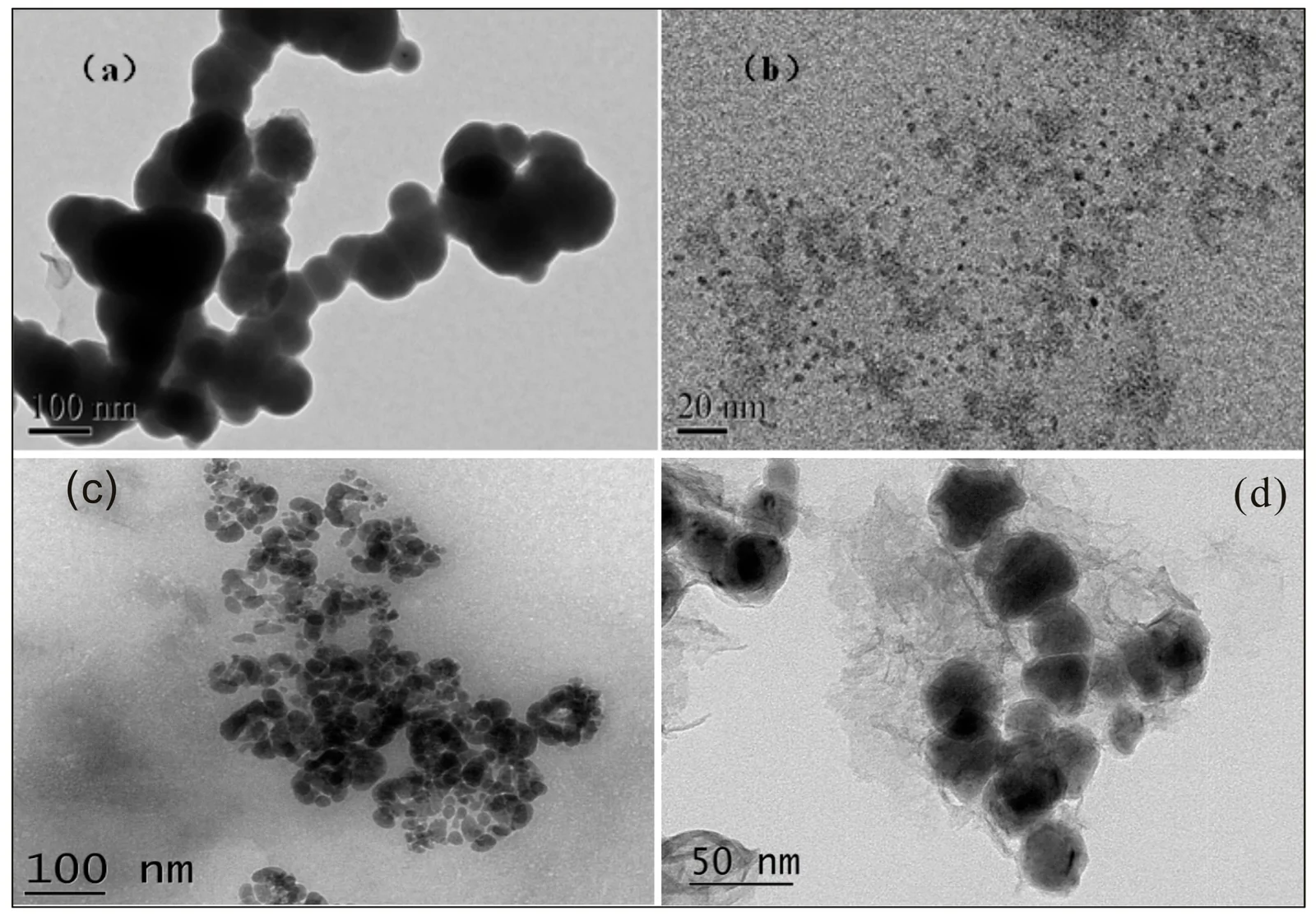
TEM images of (**a**) nZVI, (**b**) C-nZVI, (**c**) P-nZVI, and (**d**) S-nZVI.

**Figure 3:**
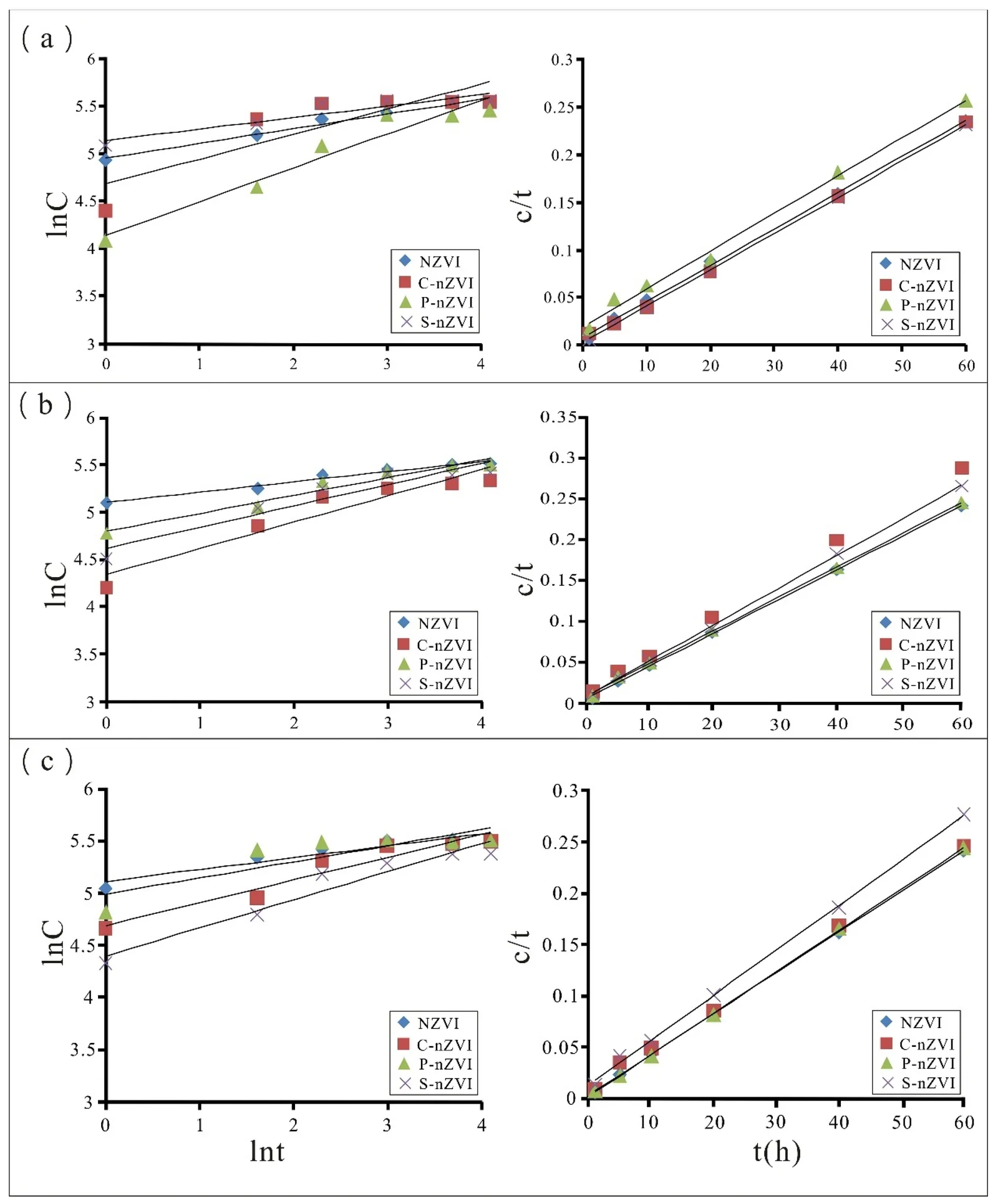
Fitting curves of the pseudo-first-order (lnC–t) and pseudo-second-order (t/C–t) kinetic models for Fe retention in soils (**a**) S1, (**b**) S2, and (**c**) S3 treated with different nZVIs.

**Figure 4:**
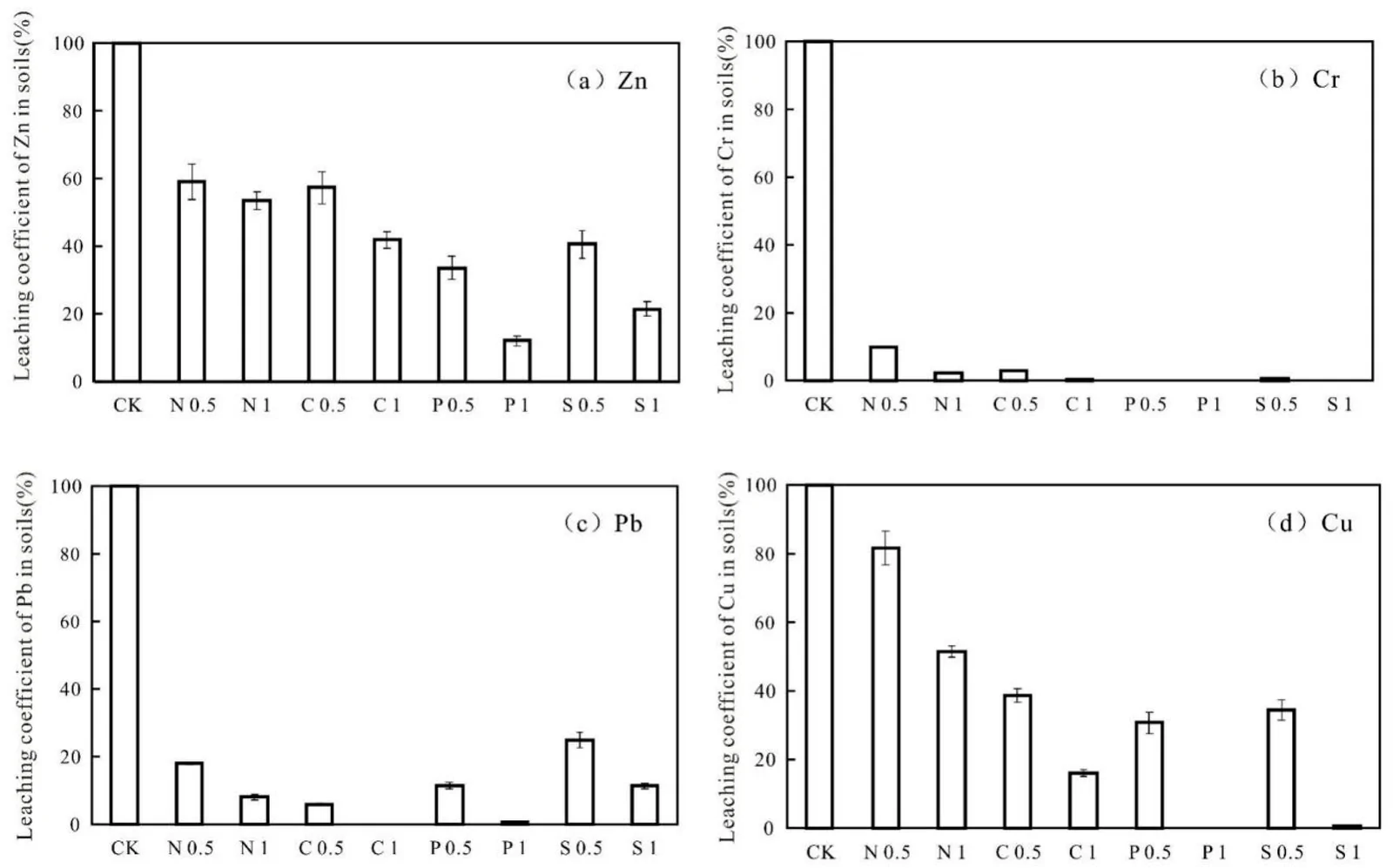
Effects of different treatments on the leaching toxicity of (**a**) Zn, (**b**) Cr, (**c**) Pb, and (**d**) Cu in soil S1. CK, untreated soil control; N, nZVI; C, C-nZVI; P, P-nZVI; S, S-nZVI. The numbers 0.5 and 1 indicate doses of 0.5 and 1 g/L Fe, respectively.

**Figure 5:**
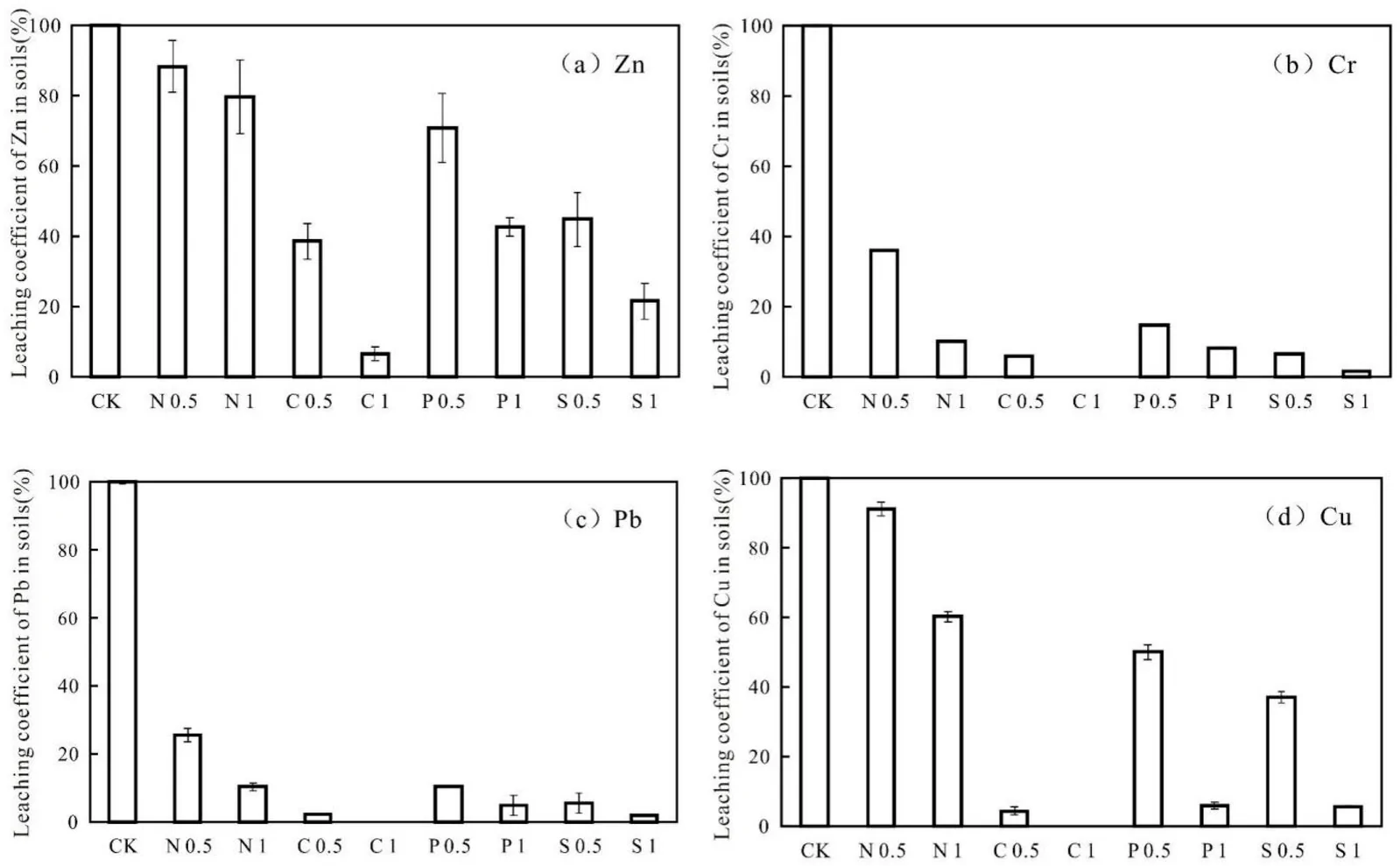
Effects of different treatments on the leaching toxicity of (**a**) Zn, (**b**) Cr, (**c**) Pb, and (**d**) Cu in soil S2.

**Figure 6:**
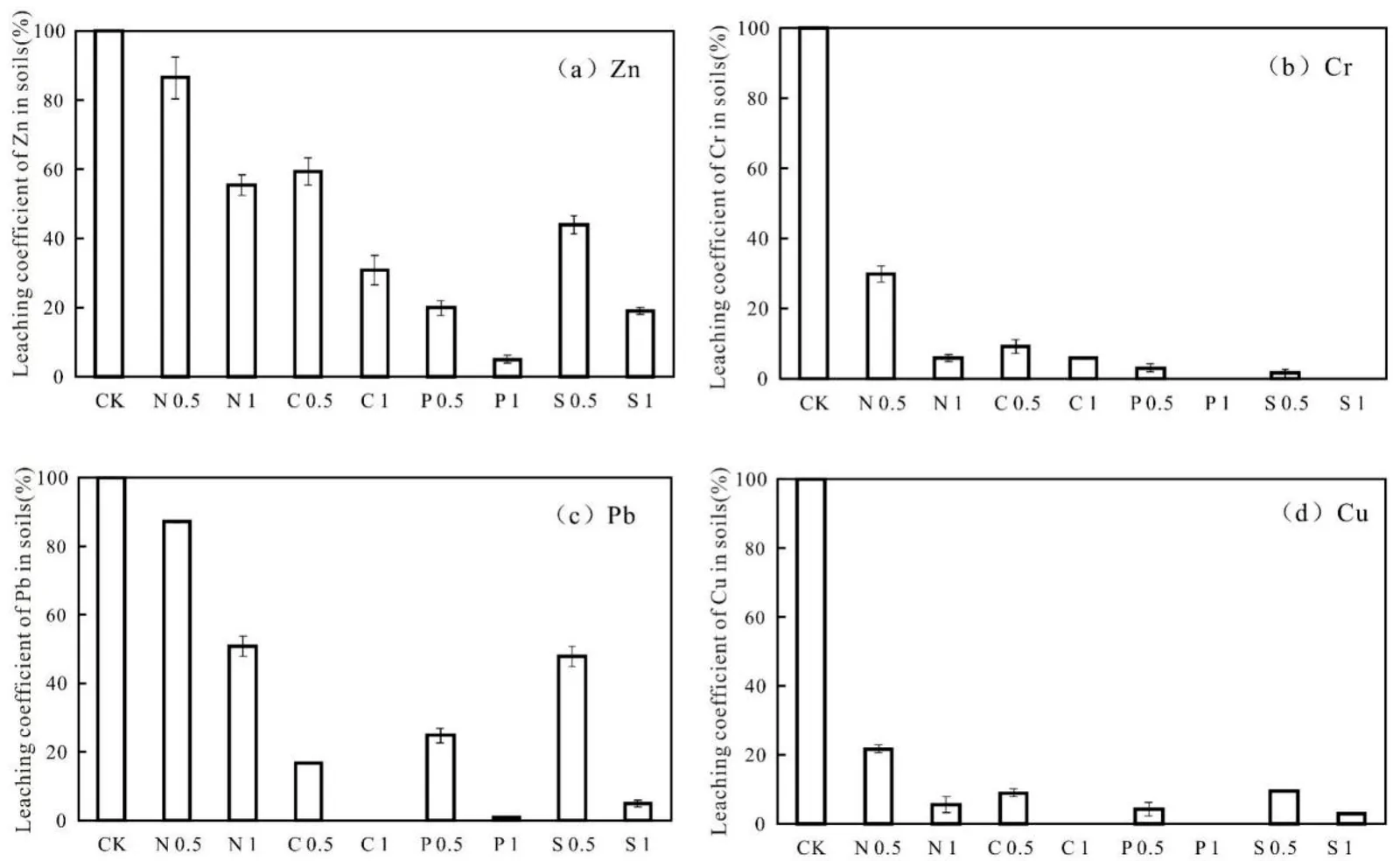
Effects of different treatments on the leaching toxicity of (**a**) Zn, (**b**) Cr, (**c**) Pb, and (**d**) Cu in soil S3.

**Figure 7:**
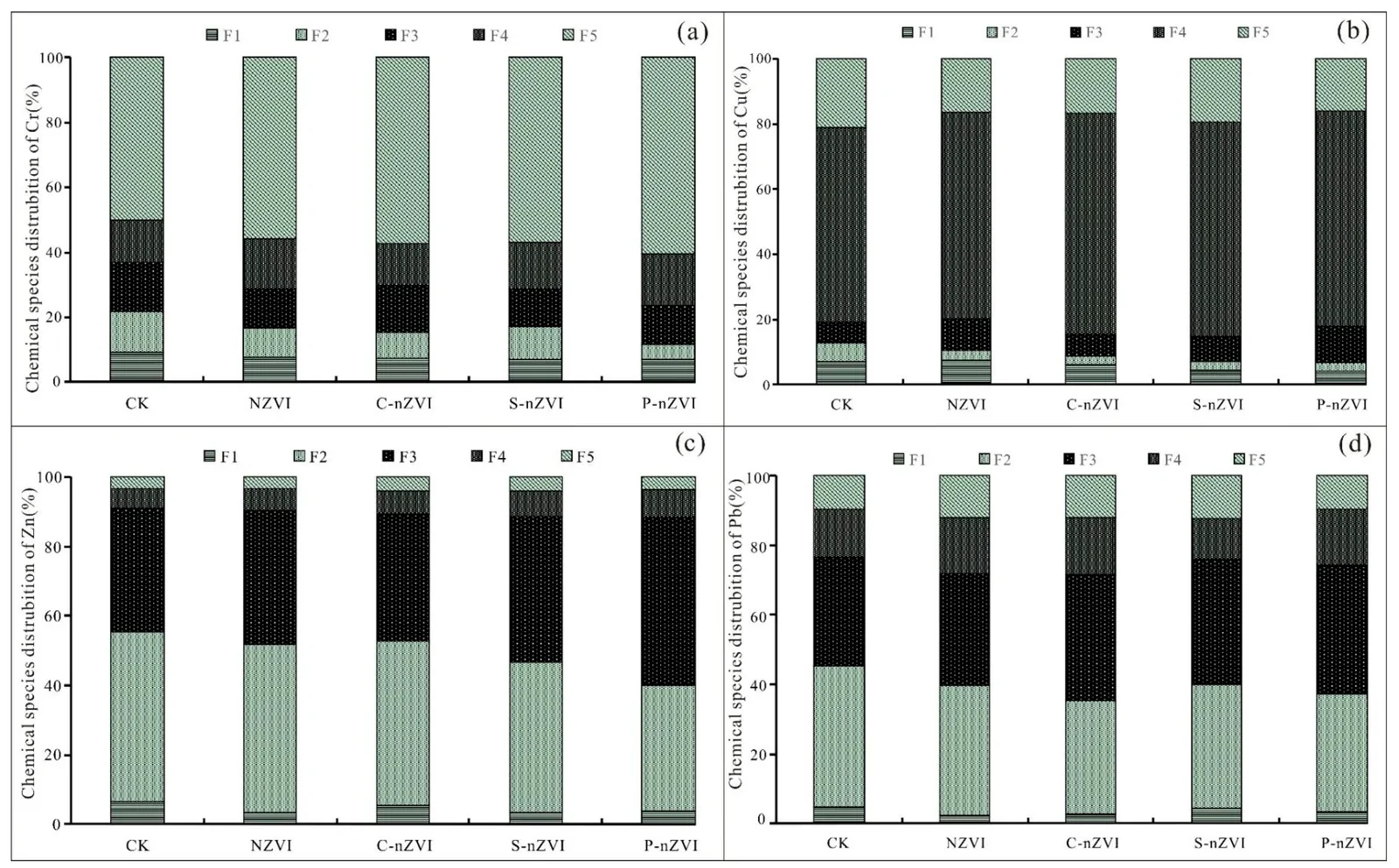
Chemical species distribution of Cr (**a**), Cu (**b**), Zn (**c**), and Pb (**d**) in soil S1.

**Figure 8:**
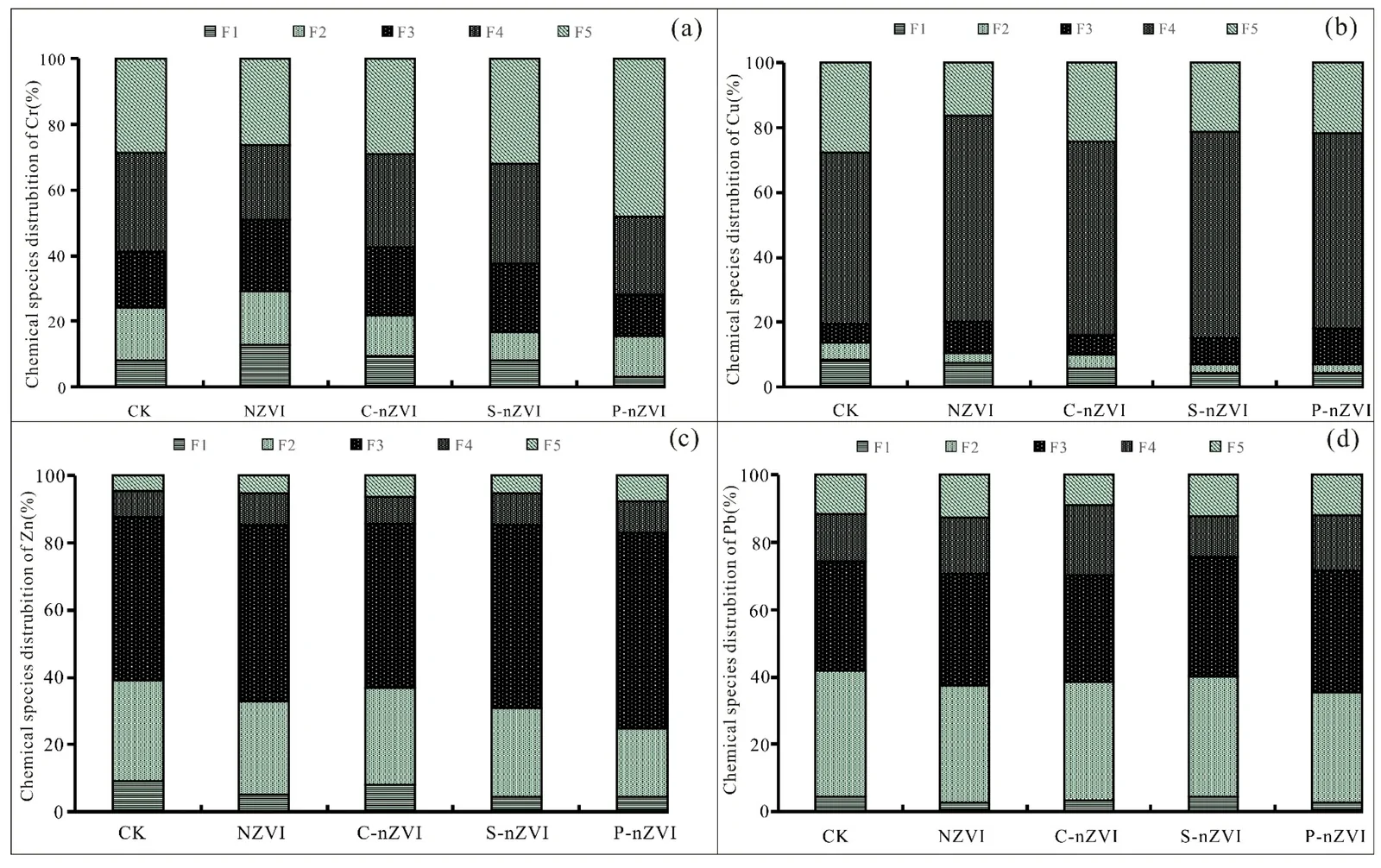
Chemical species distribution of Cr (**a**), Cu (**b**), Zn (**c**), and Pb (**d**) in soil S2.

**Figure 9:**
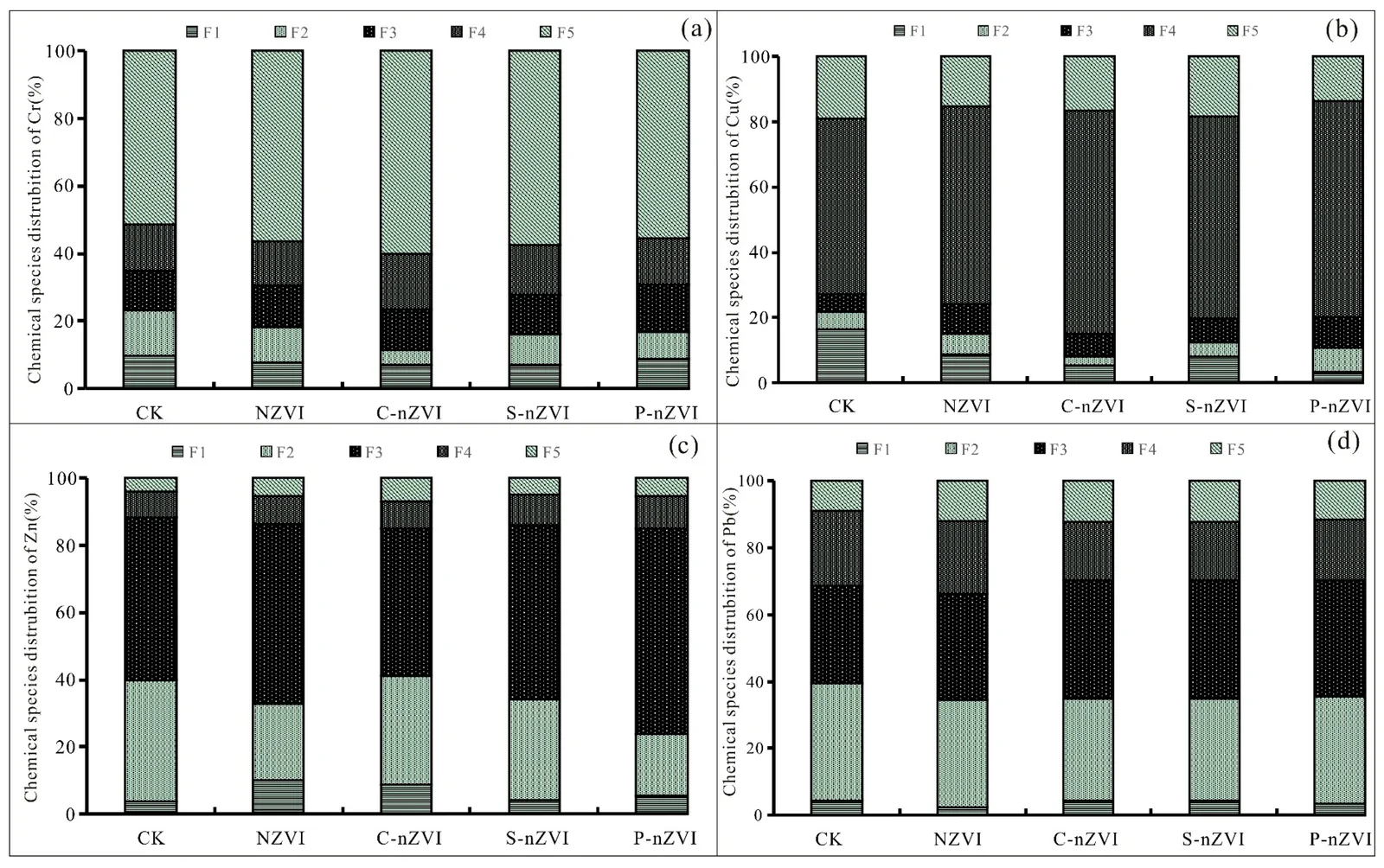
Chemical species distribution of Cr (**a**), Cu (**b**), Zn (**c**), and Pb (**d**) in soil S3.

**Table 1:** Iron content of the column effluent at different injection periods (mg/L).

Soils	Period	Iron Content (mg·L^−1^)				
		CK	NZVI	C-nZVI	P-nZVI	S-nZVI
S1	I	45.2 ± 1.59 a	69.3 ± 5.11 b	89.3 ± 3.63 c	152.7 ± 5.04 d	88.5 ± 9.03 c
	II	38.0 ± 2.34 a	51.0 ± 2.92 b	65.2 ± 4.92 c	295.2 ± 6.36 d	72.7 ± 3.58 c
	III	35.5 ± 3.75 a	42.5 ± 2.84 a	58.5 ± 4.99 b	595.5 ± 10.5 c	59.8 ± 5.65 b
S2	I	38.0 ± 2.08 b	16.1 ± 1.73 a	36.5 ± 1.87 b	77.5 ± 3.16 c	Nd
	II	37.5 ± 2.98 c	16.5 ± 2.21 b	Nd	5.30 ± 0.33 a	Nd
	III	35.0 ± 2.31 b	16.9 ± 1.92 a	Nd	Nd	123.3 ± 6.24 c
S3	I	42.0 ± 1.74 b	Nd	259.0 ± 2.25 c	12.38 ± 1.03 a	Nd
	II	37.0 ± 2.83 a	69.5 ± 3.54 b	394.4 ± 10.58 d	Nd	264.1 ± 7.25 c
	III	39.3 ± 2.16 a	63.6 ± 6.4 b	639.6 ± 12.09 d	Nd	406.8 ± 6.97 c

Data are presented as means ± standard error (n = 3). Different lowercase letters within the same row indicate significant differences among treatments (Friedman test, *p* < 0.05). Nd, not detected.

**Table 2:** Correlation coefficients between soil properties and Fe retention of nZVIs in soil.

Treatment	pH	SOM	CEC	Sand	Silt	Clay
nZVI	0.959 **	0.998 **	0.223	0.037	−0.730 *	0.932 *
C-nZVI	0.782 *	0.560 *	0.275	0.093	−0.321	0.809 *
P-nZVI	0.202	0.671 *	0.389	0.519	−0.324	0.692 *
S-nZVI	0.712 *	0.050	0.045	−0.107	0.039	0.779 *

* and ** indicate significant correlations at *p* < 0.05 and *p* < 0.01, respectively.

## Data Availability

The data presented in this study are available on request from the corresponding author.

## Abbreviations

AAS, atomic absorption spectrophotometry; BET, Brunauer–Emmett–Teller; C-nZVI, carboxymethyl cellulose-modified nano zero-valent iron; CEC, cation exchange capacity; CMC, carboxymethyl cellulose; DNAPL, dense non-aqueous phase liquid; F1, exchangeable fraction; F2, carbonate-bound fraction; F3, Fe/Mn oxide-bound fraction; F4, organic matter-bound fraction; F5, residual fraction; FE-SEM, field emission scanning electron microscopy; HR-TEM, high-resolution transmission electron microscopy; ICP-MS, inductively coupled plasma mass spectrometry; nZVI, nano zero-valent iron; P-nZVI, polyvinylpyrrolidone-modified nano zero-valent iron; PVP, polyvinylpyrrolidone; S-nZVI, sodium dodecyl sulfate-modified nano zero-valent iron; SDS, sodium dodecyl sulfate; SOM, soil organic matter; TCE, trichloroethylene; TCLP, toxicity characteristic leaching procedure; TEM, transmission electron microscopy; XRD, X-ray diffraction.
